# Analysing the effect of gender on the human–machine interaction in level 3 automated vehicles

**DOI:** 10.1038/s41598-022-16045-1

**Published:** 2022-07-08

**Authors:** Shuo Li, Phil Blythe, Yanghanzi Zhang, Simon Edwards, Weihong Guo, Yanjie Ji, Paul Goodman, Graeme Hill, Anil Namdeo

**Affiliations:** 1 School of Engineering, Newcastle University, Cassie Building, Claremont Road, Newcastle upon Tyne, NE1 7RU UK; 2 Jiangsu Key Laboratory of Urban ITS, Jiangsu Province Collaborative Innovation Centre of Modern Urban Traffic Technologies, School of Transportation, Southeast University, Southeast University Road 2, Nanjing, 211189 China; 3 Department of Geography and Environmental Sciences, Northumbria University, Ellison Place, Newcastle upon Tyne, NE1 8ST UK

**Keywords:** Engineering, Civil engineering

## Abstract

The emergence of the level 3 automated vehicles (L3 AVs) can enable drivers to be completely disengaged from driving and safely perform other non-driving related tasks, but sometimes their takeover of control of the vehicle is required. The takeover of control is an important human–machine interaction in L3 AVs. However, little research has focused on investigating the effect of gender on takeover performance. In order to fill this research gap, a driving simulator study with 76 drivers (33 females and 43 males) was conducted. The participants took over control from L3 AVs, and the timing and quality of takeover were measured. The results show that although there was no significant difference in most of the measurements adopted to quantify takeover performance between female and male. Gender did affect takeover performance slightly, with women exhibited slightly better performance than men. Compared to men, women exhibited a smaller percentage of hasty takeovers and slightly faster reaction times as well as slightly more stable operation of the steering wheel. The findings highlight that it is important for both genders to recognise they can use and interact with L3 AVs well, and more hands-on experience and teaching sessions could be provided to deepen their understanding of L3 AVs. The design of the car interiors of L3 AVs should also take into account gender differences in the preferences of users for different non-driving related tasks.

## Introduction

Automated cars potentially decrease traffic accidents, collisions and congestion, reduce emission and increase road efficiency^1,2^. It may also increase social inclusion by enhancing the mobility of older people, people incapable of driving and those with disabilities^1,3^. Vehicle automation could be graded into several levels^3–6^, each of which has different features and supports the driver in different ways. Automated vehicles equipped with level 3 automated driving systems (L3 AVs) potentially change the driver’s role significantly. L3 AVs are able to perform full dynamic driving and the driver must be present but is permitted to be fully disengaged from driving and also has the freedom to perform various types of non-driving related activities^3–6^. In L3 AVs, human drivers’ intervention in the control of the vehicle may still be required in some situations^3–6^.

The issue of takeover is a complex process representing a predominant driver-vehicle interaction in L3 AVs^7–12^. Takeovers in L3 AVs happen due to two main reasons: the first one is that the drivers themselves have a desire to manually drive the car; the second one is that the L3 AV is encountering situations that are beyond the system capabilities, such as driving in places without complete road signage and markings, construction sites, or in rural areas with no signal or network connections, so it needs drivers to reassume the control of the car^9,10^. The takeovers launched by the human drivers are generally less challenging compared to those issued by the L3 AV systems^10,12,13^. In the L3 AV system-issued takeovers, the L3 AV system firstly senses the situation and then issues a request to the drivers to inform them about the upcoming critical situation and allow them to retake the control and deal with the situation within an enough time budget^3–7,13^. Previous research has investigated takeover control process in L3 AVs and found that the out of the loop of driving leads to deteriorated performance among drivers compared to when they were engaged in driving in L3 AVs^7,8,12–16^.

### Effects of gender on drivers’ interaction with automated vehicles

To date the demographic factor which has been well-considered in research of takeover in L3 AVs is age^12,13,17–20^. Apart from age, gender is another important demographic factor in ergonomics research^21–23^. It is also one of the most frequently adopted variables in research into driving behaviour in conventional vehicles. Gender differences in driving behaviour have been identified in terms of the number of crashes^24–26^, the patterns of crashes^26^, risk perception^27^, driving confidence^28^, the self-assessment of driving skills^29^, anger while driving and traffic violations^30^. In addition, previous research has also found marked gender differences in terms of interacting with in-vehicle systems. Edwards et al.^31^ found gender difference in relation to interactions with in-vehicle navigation systems, with older female drivers more willing to adopt landmarks to navigate their journey. Yang et al.^32^ found that men and women were affected differently when driving while interacting with in-vehicle information systems.

Given the emerging trends of vehicle automation, some studies have also considered the effect of gender when researching automated vehicles and have identified significant gender differences. Hohenberger et al.^33^ found gender difference in terms of the desire to use automated vehicles, with women perceiving less enjoyment and higher levels of concern towards automated vehicles than men. In addition, Haboucha et al.^34^ investigated drivers’ willingness to own and use automated vehicles and they found that, in Israel, men are more likely to buy or use automated vehicles compared to women. These findings were supported by Hulse et al.^35^ who investigated people’s attitudes and opinions towards automated vehicles and found significant gender differences, with female participants less accepting of automated vehicles compared to the male participants. Similarly, research by Hand and Lee^36^ revealed significant gender differences in relation to opinions and concerns about automated vehicles. They found that male drivers were more positive and more willing to own automated vehicles than female drivers. Abraham et al.^37^ found significant gender differences in terms of the methods used in learning to use in-vehicle systems and automated vehicles, with men more inclined to learn by themselves using online instructions and manuals, while women preferred to be taught. In addition, Useche et al.^38^ found gender difference on drivers’ intention to use an automated vehicle, with male users’ intentions is influenced by connectivity, fuel consumption, energy and trip efficiency and safety-related issues, while female’s intention is associated with driving demands, trip efficiency and safety features. Moreover, Muslim et al.^39^ identified significant effect of gender on people’s reaction time when interacting with automated driving systems and also reported significant interaction effect between gender and the design of the human–machine interface of the automated driving systems.

### Purpose of this study

In spite of the fact that the effects of gender on the driving behaviour of conventional vehicles are widely accepted, existing research into the takeover of the control of L3 AVs has neglected this significant demographic factor. Some studies have considered gender effects when researching automated vehicles and it has been revealed that some gender differences do exist, with female drivers seeming to be less accepting and more concerned about automated vehicles compared to male drivers^33–36^. However, these studies generally focussed on an attitudinal perspective and examined gender differences in terms of drivers’ opinions and acceptance of and concerns about automated vehicles. Research investigating gender differences in actually interacting with automated vehicles from a performance perspective is still limited. Knowledge regarding the impact of gender on drivers’ takeover performance in L3 AVs still remains unclear. Such knowledge could be important in the design of user-friendly L3 AVs, and thereby seems crucial in ensuring their usability. The lack of such knowledge could not only potentially reduce the benefits that L3 AVs are expected to deliver, but could also potentially have a negative impact in the light of the significance of gender roles in society. In addition, Neglecting the impact of gender potentially lead to serious issues in terms of transport equality^40,41^.

Therefore, the purpose of this study is to investigate the effect of gender on drivers’ takeover performance in L3 AVs. The knowledge yielded by this study could potentially inform the design of L3 AVs and facilitate safer and smoother human–machine interactions in L3 AVs for potential end-users from different demographic groups.

## Method

### Participants

To be eligible to participate in this study, a participant was required to have a valid UK driving licence; to be an active driver at the time they participated in the study; meets the minimum eyesight standard for driving in the UK; with normal or correct hearing; do not experience motion sickness; to be fluent in English. A total of 76 participants participated in the study who were aged between 20 and 81 years (mean = 49.21 years, SD = 23.32 years). They were recruited in Newcastle upon Tyne. Table 1 shows their annual driving mileages by gender group. The 33 subjects who were female drivers aged between 20 and 81 years (mean = 47.73 years, SD = 24.15 years), among them, 17 were younger female drivers (aged between 20 and 35 years, mean = 25.12 years, SD = 4.55 years) and 16 were older female drivers (aged between 60 and 81 years, mean = 71.75 years, SD = 5.26 years). And 43 were male drivers aged between 21 and 79 years (mean = 50.35 years, SD = 22.88 years), among them, 20 were younger male drivers (aged between 21 and 35 years, mean = 26.85 years, SD = 4.34 years), 23 were older male drivers (aged between 61 and 79 years, mean = 70.78 years, SD = 6.65 years). There was no significant difference in the distribution of younger and older participants inside the two gender groups, as assessed using a Chi-square test, *X*^2^(7) = 8.290, p = 0.308.

**Table 1:** Annual mileage of participants by gender.

Annual mileage (miles)	0–3000	3000–6000	6000–10,000	10,000–15,000	15,000+	Total
Female drivers	11	13	7	2	0	33
Male drivers	10	10	10	10	3	43
Total	21	23	17	12	3	76

### Apparatus

**Figure 1:**
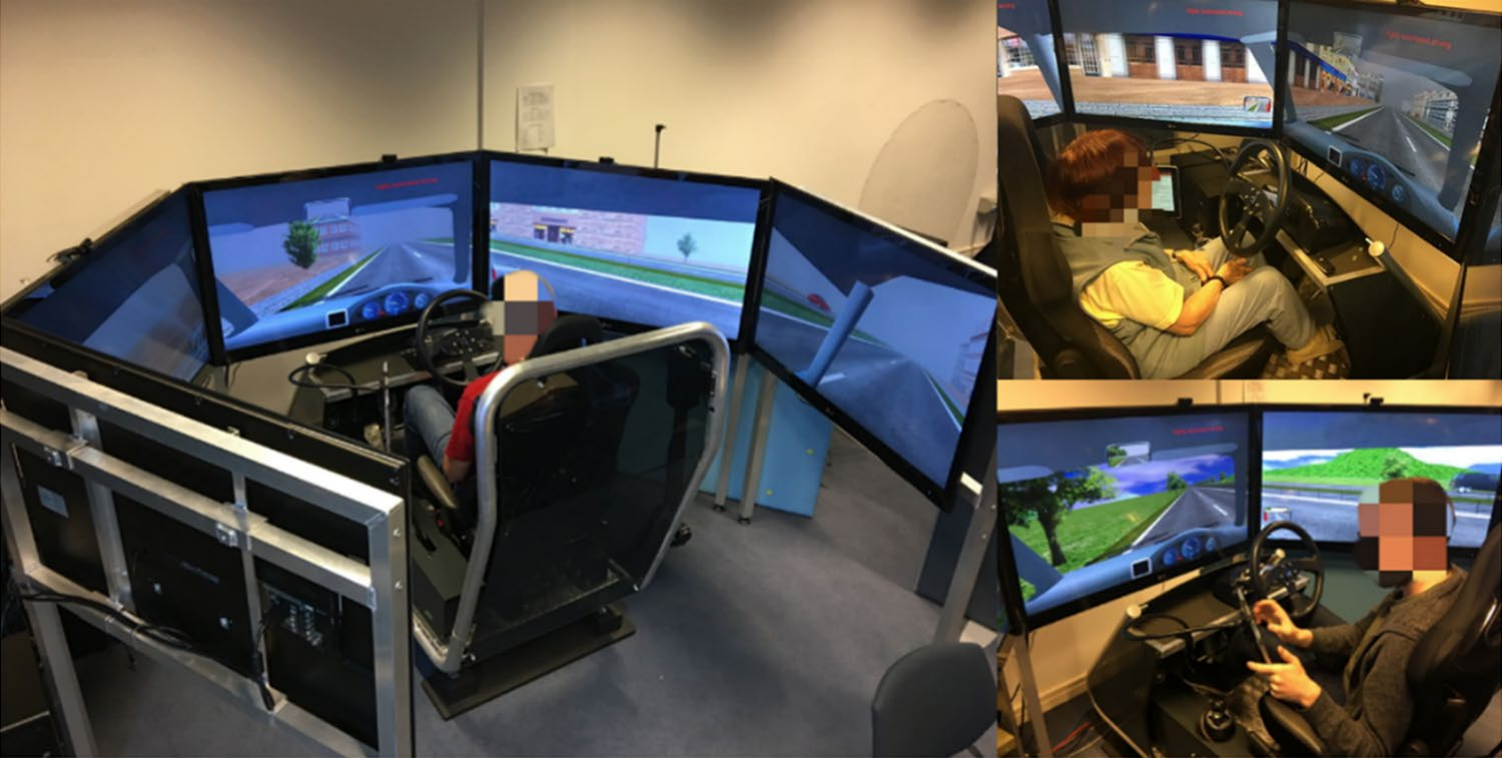
Newcastle University Fixed-based ST software Jentig50 driving simulator.

This study was taken place at the driving simulator laboratory of Newcastle University, as shown in Fig. 1. It is featured the simulated driving scene displayed by five 50-in. 1080p LCD screens connected by a metal framework. It is also equipped with all the vehicle controls, including foot controls: accelerator, brake and clutch pedals and hand controls: a dynamic force feedback steering wheel, gears, a handbrake and indictors as well as an adjustable driver seat. It has a simulated dashboard, with rear-view and side mirrors on the screens. Sound is played via a 5.1 surround-sound system which enables a real-life driving experience.

### L3 AV scenario on the driving simulator

**Figure 2:**
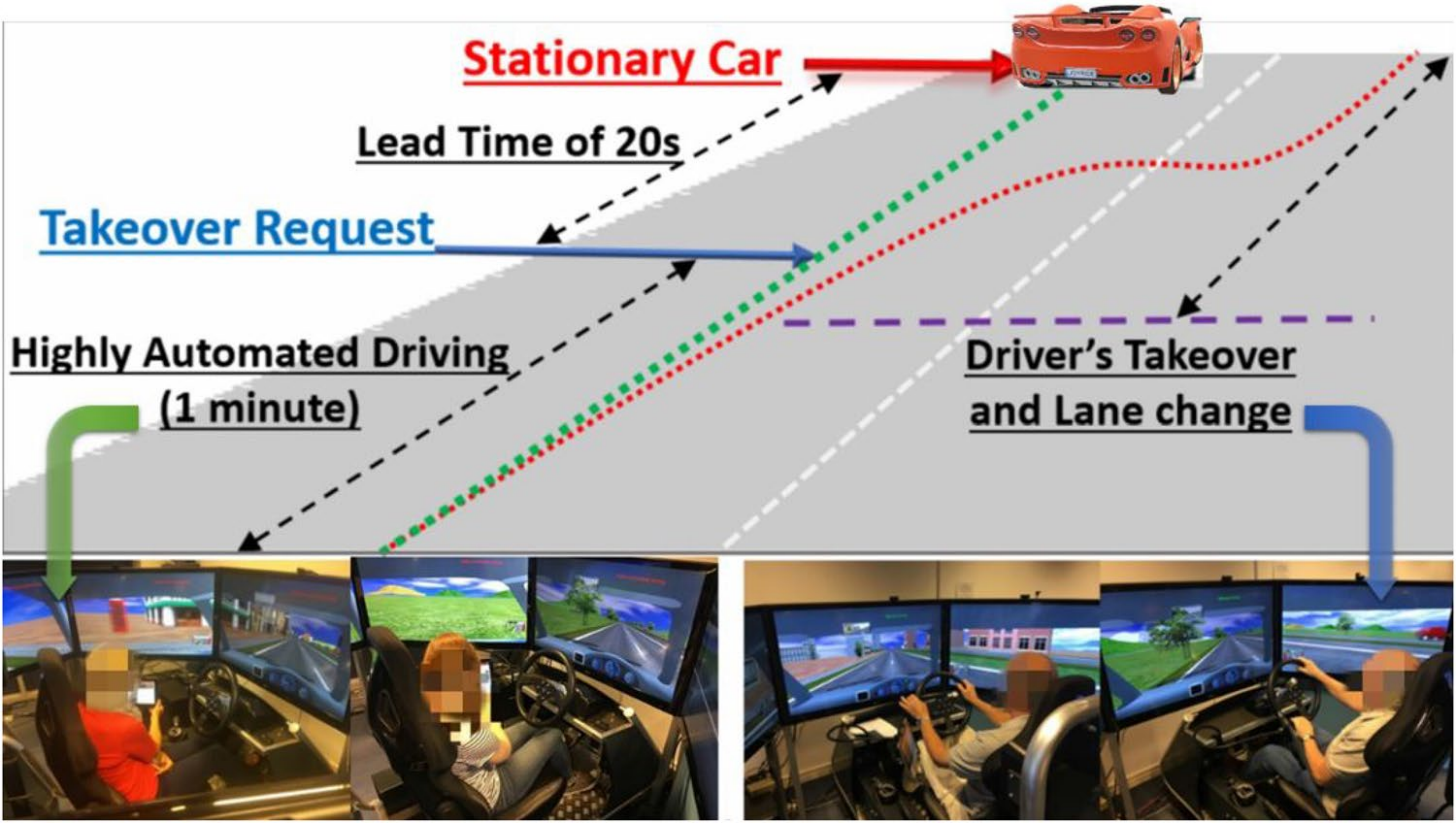
Illustration of the L3 AV scenario.

**Figure 3:**
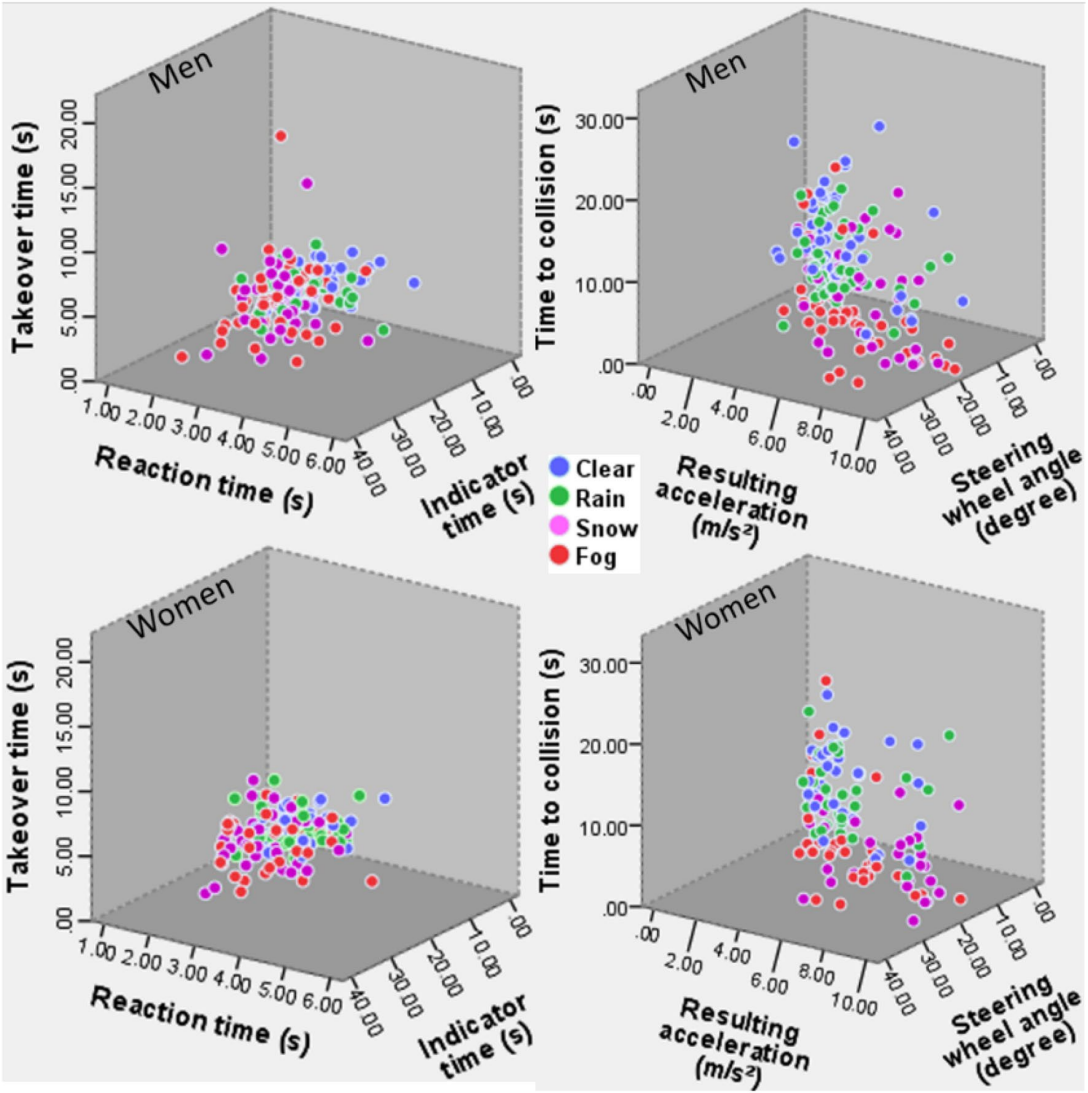
3D scatterplots of time aspects of takeover and takeover quality for female and male drivers.

As Fig. 2 illustrates, the L3 AV scenario starts with a 1-min automated driving. During this portion of time, the L3 AV is fully responsible for driving the car and drivers are allowed to be completely out of the driving loop and to perform a reading task from a tablet mounted on the 45° left-hand side of the steering wheel. At the 1-min mark, the L3 AV system senses a stationary red car suddenly blocking the lane ahead, and it then issues a visual and auditory takeover request to the driver and keeps driving at its current speed. The takeover request consists of a red message (approximate font size 42 mm) on the screen (located on the right side next to the simulated rear-view mirror) reading ‘Please take over’ and a computer-generated female voice saying ‘Attention! Please take over the vehicle control’. Predominantly red message was used to represent high criticality. Drivers should then stop reading, take over control of the car and deal with the red car ahead by changing to the next lane within a time budget of twenty. After they have avoided the red car they keep driving till they were asked to pull over and the scenario ends^12,17,18^. Two kinds of road are used for the L3 AV scenario in this study: a city road and a motorway (see Fig. 3). Two common UK national speed limits were applied (30 and 60 mph). The L3 AV scenario runs in four weather conditions-clear, rain, snow and fog, with the visibility of 1000 m, 400 m, 200 m and 100 m, respectively^17^.

### Experimental design

When designing the experiment in this study, several independent variables were considered, including age (younger, older), gender (female, male), and weather (clear weather, rain, snow and fog). Two separate pieces of research previously published by the present authors have reported on the effects of age and weather^17^ as well as issues of age and driving disengagement in L3 AVs^12^. Since the data did not yield any significant interactions between gender and age as well as gender and weather, the effects of gender were decided to be reported as separate piece of research^17^. Therefore, the present study only focusses on the influence of gender which is a between-subjects independent variable consisting two levels: Female and Male.

As shown in Table 2, several dependent variables were adopted to quantify participants’ takeover performance and attitude.

**Table 2:** Overview of the dependent variables.

Dependent variables	Definition	Data collection	Data type	Unit
Reaction time	The time between the takeover control request and drivers switching back to a safe and ready manual driving position (driver’s hands on the steering wheel, feet on the pedals and eyes on the road)	Video rating	Continuous	Seconds
Takeover time	Time between the point of a takeover request and the point that the automation system perceives an active input on the car controls from the driver (an action which changes the steering wheel by 2° and/or a movement of 10% of the accelerator or brake pedals)	Driving simulator	Continuous	Seconds
Indicator time	Time between the points the L3 AV initiates a takeover request and the point of the driver’s initiation of an indicator light signal warning fellow road users that the driver intends to change lanes to avoid the stationary red vehicle ahead	Driving simulator	Continuous	Seconds
Time to collision (TTC)	Time required for the automated vehicle to crash into the stationary car ahead in the driving lane if it continued at its current speed at the moment or if it has completely avoided the stationary car blocking the road ahead	Driving simulator	Continuous	Seconds
Resulting acceleration	The maximum force that the car transfers to the ground (a square root of sum of the squares of maximum lateral and longitudinal accelerations	Driving simulator	Continuous	m/s^2^
Steering wheel angle	Standard deviation in degrees from the centre-line of the steering wheel	Driving simulator	Continuous	Degrees
Hasty takeover	Any abrupt takeover where a drivers’ takeover time is smaller than the reaction time	Driving simulator	Nominal	Count
Collisions and critical encounters (CCE)	Any takeover with a minimum TTC of less than 1.5 s	Driving simulator	Nominal	Count
Reaction type	Type of reaction strategy driver adopts in response to the stationary car ahead (steering only or steering and braking	Driving simulator	Nominal	Count

### Experimental procedure

This research was implemented using the following procedure^12,17^. Firstly, the researcher met the participants at the entrance of the Newcastle University driving simulator laboratory and guided them to the lab. Then their driving licenses were checked and they filled the ethical form and demographic questionnaire. After that, the purpose of the study was verbally explained to them, which was to examine their performance in terms of timing and quality when taking over control with level 3 automated vehicles in different weather and road conditions. All subjects were given sufficient time to become familiar and comfortable with the driving simulator. During this session, they firstly conducted a number of manual driving sessions to enable them to become familiar with the vehicle controls on the driving simulator; secondly, they practiced to become familiar with the required physically disengagement position (hands off the steering wheel and feet off the pedals) in the level 3 automated driving mode; Also, they were trained to perform the required non-driving related task in the level 3 automated driving, which is a read-aloud task using a tablet installed on the 45° left to the central line of the steering wheel^12,17^; and finally, they were trained to execute the resuming vehicle control action by stopping the read-aloud task and switching from the physically disengagement position to the normal manual driving position. This session stops until they verbally indicated that they were ready and would like to start the experiment. They were informed that their driving performance in all sessions would be evaluated; they were required to reassume control of the car as soon as they perceived the takeover request initiated by the level 3 automated driving system. After they had taken over the control of the car, they should not exceed the speed limit, and keep driving until they were told to stop, use indicator signals when changing lane, and drive as they do in their daily life. They were told that they could withdraw from the study at any time. After that, the sessions started. Drivers were asked to drive several sessions differentiated by weather. The order of the driving sessions was randomised so as to avoid the order effects. The data of participants’ takeover performance was collected by the driving simulator with a frequency of data collection of 20 samples/s. The analysis of data was conducted using SPSS.

## Results

### Overall takeover performance and reaction type

Figure 3 illustrates the overall takeover performance for female and male drivers in different weather conditions. It shows that compared to in clear weather condition, both female and male drivers exhibited slower time-aspects of takeover and worse takeover quality in adverse weather conditions.

**Figure 4:**
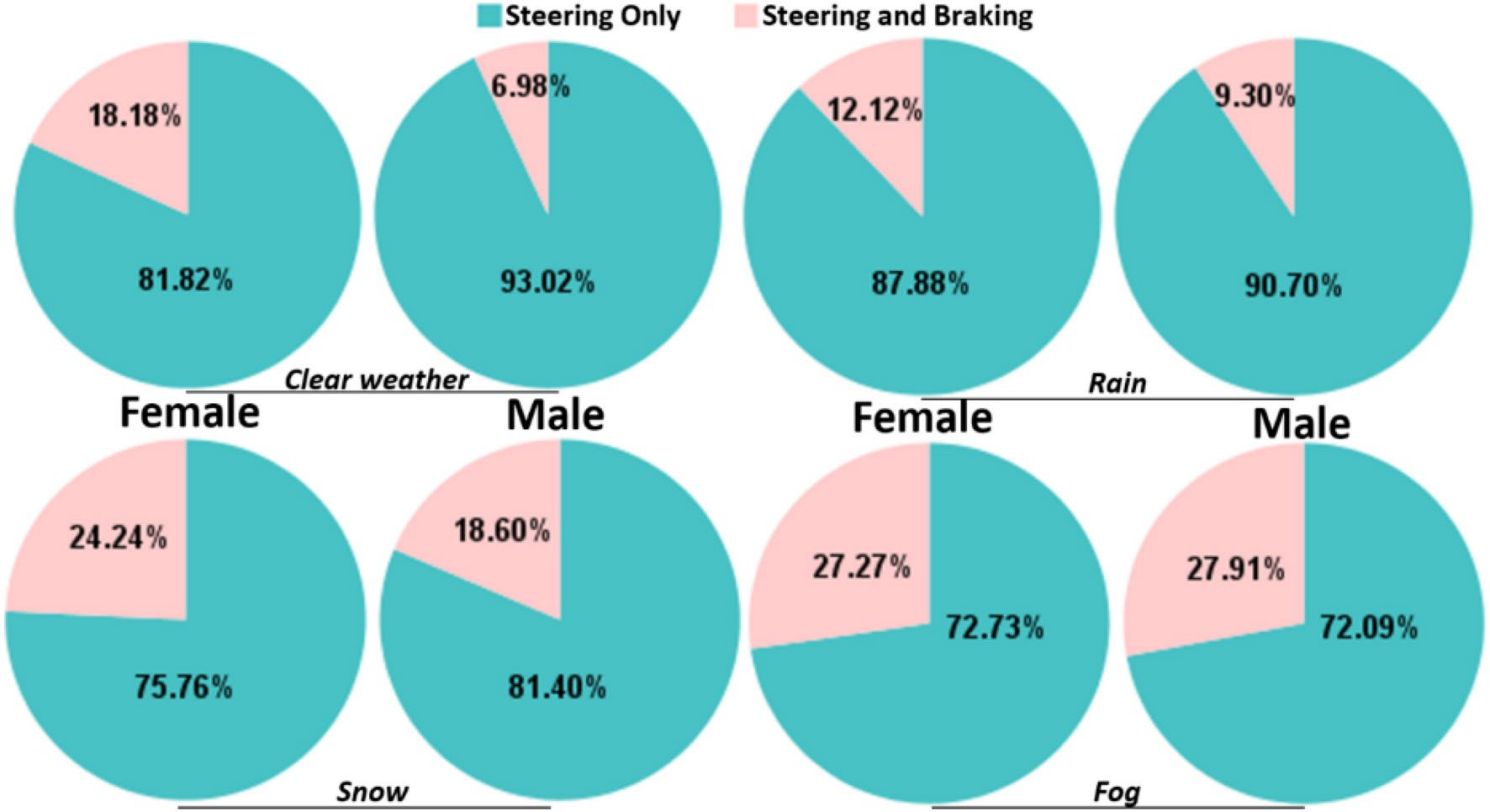
Proportion of reaction types for female and male drivers in different weather conditions.

And the proportion of reaction types by different gender groups during takeover in the L3 AVs in different weather conditions is showed in Fig. 4. In the clear weather condition, 18.18% of female drivers (n = 6) reacted to the stationary red car by steering and braking, and 81.82% of them (n = 27) reacted by steering only. However, for male drivers, only 6.98% (n = 3) reacted by steering and braking, and 93.02% (n = 40) reacted by steering only. There was no significant difference in the reaction type between female and male drivers in clear weather as assessed using a Chi-square test, *X*^2^(1) = 2.245, p = 0.134. In the rain condition, for the female drivers, 12.12% (n = 4) responded by steering and braking, and 87.88% (n = 29) by steering only. For male drivers, 9.30% (n = 4) responded by steering and braking, and 90.70% (n = 39) by steering only. There was no significant difference between female and male drivers’ reaction type, *X*^2^*(*1) = 0.158, p = 0.691. In the snow condition, for the female drivers, 24.24% (n = 8) reacted by steering and braking, and 75.76% (n = 25) reacted by steering only. For the male drivers, 18.60% (n = 8) reacted by steering and braking, and 81.40% (n = 35) by steering only, where the difference in reaction type between the males and females was not significant, X^2^(1) = 0.357, p = 0.550. In the fog condition, for the female drivers, 27.27% (n = 9) responded by steering and braking, and 72.73% (n = 24) by steering only. For the male drivers, 27.91% (n = 12) responded by steering and braking, and 72.09% (n = 31) by steering only, and the difference in reaction type between males and females was again not significant, X^2^(1) = 0.004, p = 0.951.

### Hasty takeover

**Figure 5:**
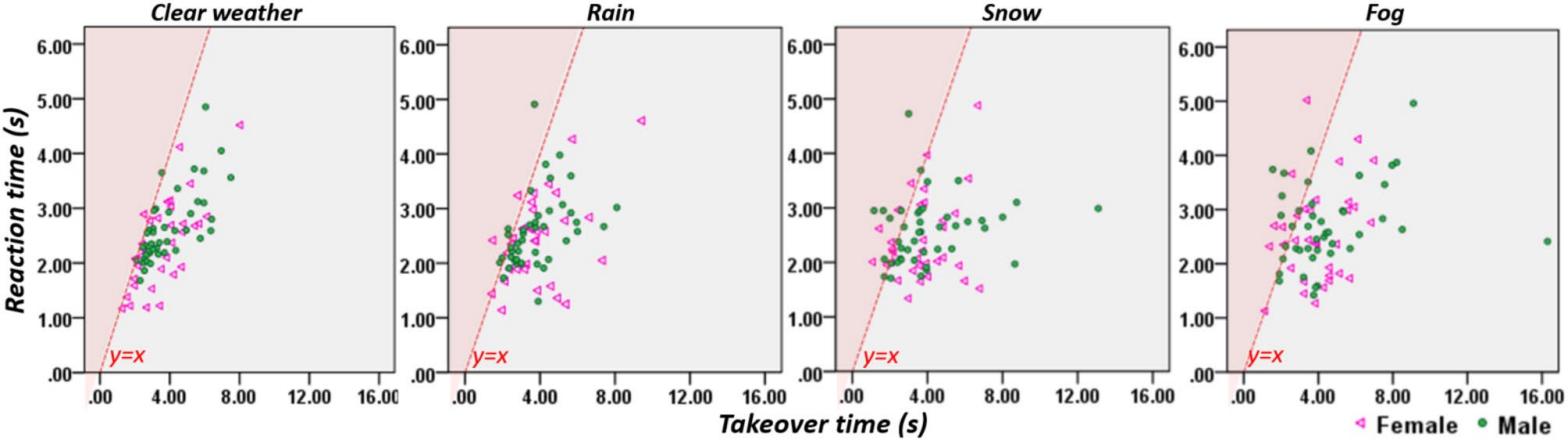
Illustration of hasty takeovers of different gender groups using scatterplot of reaction time and takeover time.

Figure 5 illustrates the data on hasty takeovers that female and male drivers exhibited when taking over control of the vehicle under different weather conditions, using scatterplots of reaction time and takeover time. The red dotted lines in Fig. 5 are y = x. If a data point falls on the left-hand side of the y = x line (the highlighted red area), this suggests the driver had a longer reaction time than takeover time. Drivers of this type generated active input to the vehicle before they had completely switched to the position that is ready for manual driving and therefore this could reflect hasty takeover behaviour.

As Table 3 shows, in total, female drivers exhibited 17 hasty takeovers and the male drivers had 23. However, the difference was not significant, as assessed by a Chi-square test, X^2^(1) = 0.016, p = 0.900. In the clear weather condition, 3 drivers exhibited hasty takeovers, including 2 female and 1 male drivers. A Chi-square test revealed that the difference between the women and men in the number of hasty takeovers is not significant, *X*^2^(1) = 0.687, p = 0.407. In the rain condition, 7 hasty takeovers were recorded, including 2 by female drivers and 5 by male drivers. However, the difference in hasty takeovers between women and men is not significant, *X*^2^(1) = 0.692, p = 0.405. In the snow condition, 15 hasty takeovers were recorded, including 7 by female drivers and 8 by male drivers. The difference in the hasty takeover between two gender groups was once again not significant, *X*^2^(1) = 0.080, p = 0.777. Finally, in the fog condition, 15 drivers exhibited hasty takeovers, including 6 by female drivers and 9 by male drivers, and the difference is not significant as assessed by a Chi-square test, *X*^2^(1) = 0.089, p = 0.765.

**Table 3:** Number of hasty takeovers by male and female drivers in different weather conditions.

	Clear	Rain	Snow	Fog	Overall
	Hasty takeover	Hasty takeover	Hasty takeover	Hasty takeover	Hasty takeover
Female	2	2	7	6	17
Male	1	5	8	9	23
Total	3	7	15	15	40

### Collisions and critical encounters (CCEs)

**Figure 6:**
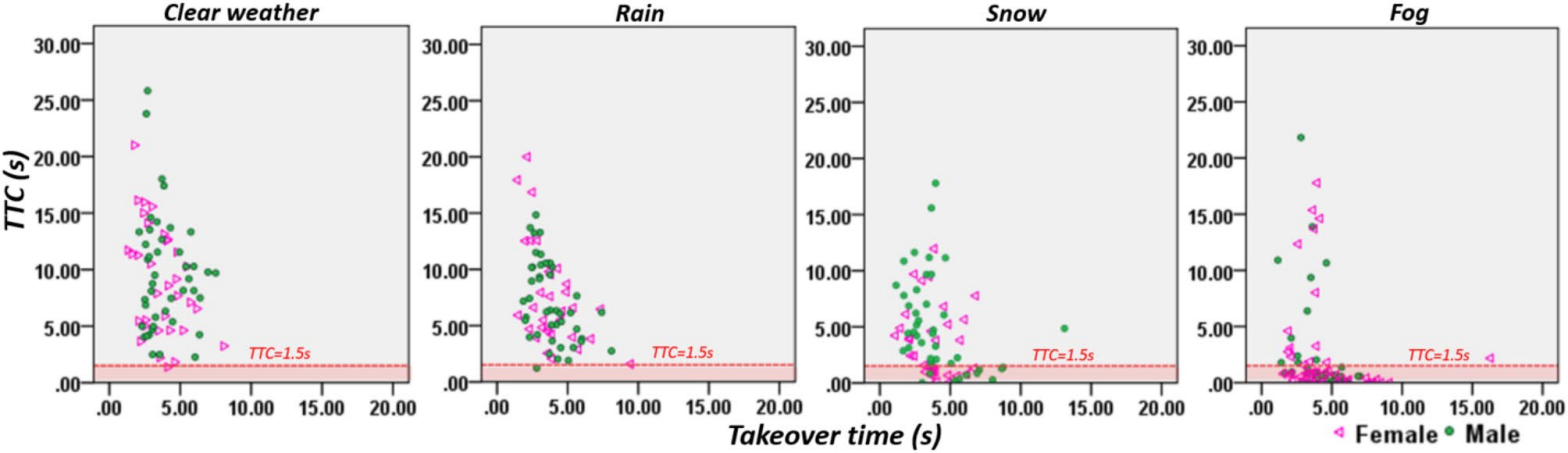
Illustration of CCEs of different gender groups using scatterplot of TTC and takeover time.

Figure 6 illustrates the numbers of CCEs that female and male drivers exhibited when taking over the control of the vehicle under different weather conditions, using scatterplots of TTC and takeover time. The red dotted lines in Fig. 6 are where y = 1.5 (TTC = 1.5 s), and if a data point falls below the y = 1.5 line (the highlighted red area), it represents a collision or a critical encounter, where the latter is defined as any takeover with a minimum TTC of less than 1.5 s^35^. In total, 40 CCEs were recorded, including 17 by female drivers and 23 by male drivers. Again, the difference in CCEs between female and male drivers is not significant, *X*^2^*(1)* = 0.016, p = 0.900.

As Table 4 shows, in the clear weather condition, only 1 CCE was recorded, it is a female driver. The difference in CCEs between the two gender groups is not significant, X^2^(1) = 1.320, p = 0.251. In the rain weather condition, there was also 1 CCE recorded, which was for a male driver. The difference in CCEs between the female and male drivers is not significant, X^2^(1) = 0.778, p = 0.378. In the snow weather condition, 23 CCEs are recorded, 13 of female drivers and 10 of male drivers, however the difference in the CCEs between the two gender groups is not significant, X^2^(1) = 2.304, p = 0.129. Finally, in the fog weather, 50 CCEs were recorded, 21 for female drivers and 29 for male drivers, and the difference in CCEs between the two gender groups is not significant X^2^(1) = 0.120, p = 0.729.

**Table 4:** The CCEs of female and male drivers in different weather conditions.

	Clear	Rain	Snow	Fog	Overall
	CCE	CCE	CCE	CCE	CCE
Female	1	0	13	21	35
Male	0	1	10	29	40
Total	1	1	23	50	75

### Reaction time

**Figure 7:**
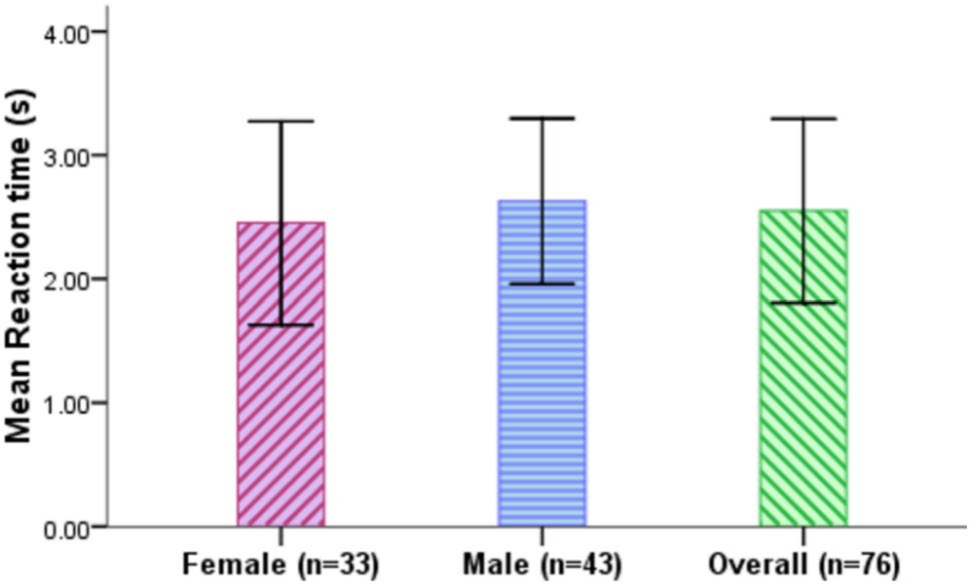
Reaction time for female and male drivers (Error bars =  ± SD).

Reaction time is the time between the time point that L3 AV issues the takeover request to the driver and the point that that the driver has changed to the position of being ready for manual driving. It measures how quickly drivers react to the request to take over control of the L3 AV. Figure 7 and Table 5 show the mean reaction times that female and male drivers exhibited when taking over control from the L3 AV. Overall the participants had a mean reaction time of 2.55 s (SD = 0.74 s). Female drivers exhibited a mean reaction time of 2.45 s (SD = 0.82 s), while the male drivers had a mean reaction time of 2.63 s (SD = 0.67 s).

**Table 5:** Descriptive analysis of reaction time.

Reaction time (s)					
	Mean	SD	Min	Max	95% CI
Female	2.45	0.82	1.13	5.02	2.31–2.59
Male	2.63	0.67	1.30	4.96	2.53–2.73
Total	2.55	0.74	1.13	5.02	2.47–2.63

An Independent samples t-test was adopted to examine whether or not there is a significant difference in reaction times between female and male drivers. The results showed that gender has a statistically significant effect on reaction time, t(248.695) = − 1.991, p = 0.048, Cohen's d = 0.240 with female drivers (M = 2.45 s, SD = 0.82) exhibiting faster reaction time compared to the male drivers (M = 2.63, SD = 0.67), with a significant difference of 0.17 s (95% CI 0.002 s to 0.35 s).

### Takeover time

**Figure 8:**
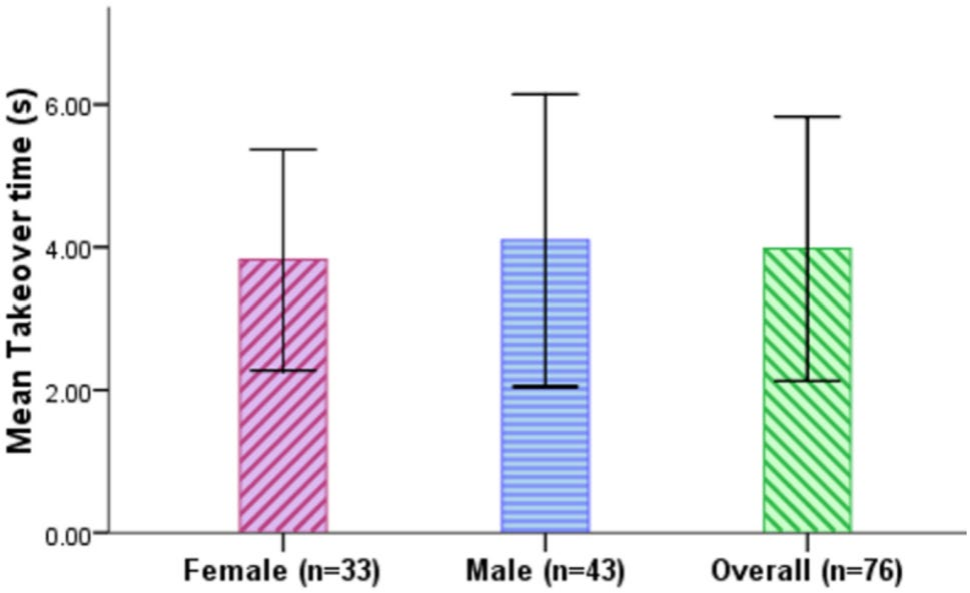
Takeover time for female and male drivers (Error bars =  ± SD).

Takeover time is the time between the time point that the L3 AV initiates the takeover request to the drivers and the point that drivers execute their first active input to the L3 AV. It measures how quickly drivers gain control of the vehicle when requested to do so by the L3 AV. Figure 8 and Table 6 show that, overall, the participants exhibited a mean takeover time of 3.98 s (SD = 1.85 s). Female drivers had a slightly faster takeover (m = 3.82 s, SD = 1.55 s) than the male drivers (m = 4.10 s, SD = 2.05 s). In order to examine whether or not the difference in takeover times between the two gender groups is significant, an Independent samples t-test was implemented. It shows that the difference is not statistically significant, t(302) = − 1.275, p = 0.203.

**Table 6:** Descriptive analysis of takeover time.

Takeover time (s)					
	Mean	SD	Min	Max	95% CI
Female	3.82	1.55	1.10	9.45	3.56–4.09
Male	4.10	2.05	1.15	16.30	3.79–4.40
Total	3.98	1.85	1.10	16.30	3.77–4.19

### Indicator time

**Figure 9:**
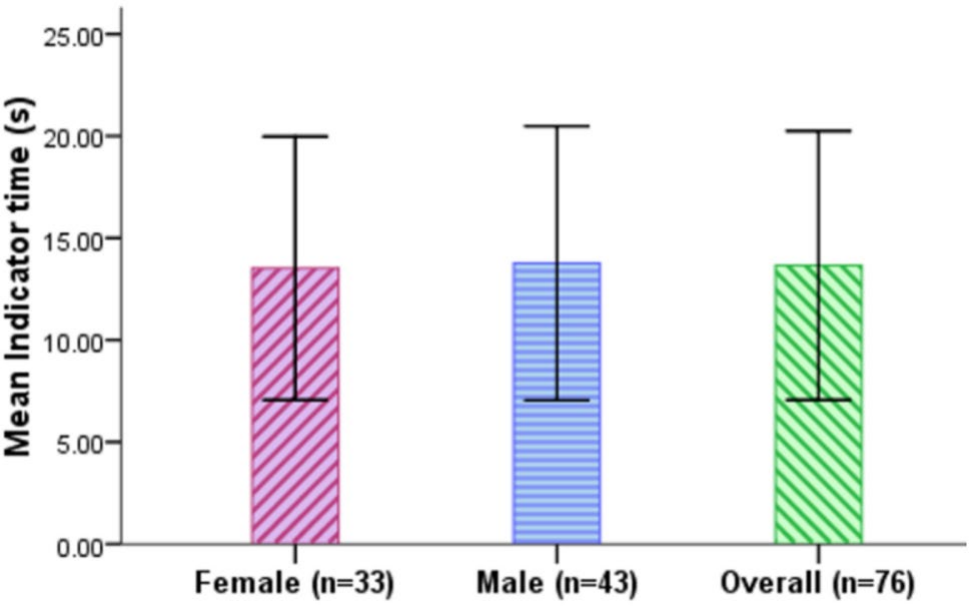
Indicator time for female and male drivers (Error bars =  ± SD).

Indicator time is the time between the time point that the L3 AV system sends the takeover request to the driver and the point that the driver turns on the signal to indicate the lane change. It measures how quickly drivers make the decision to change lane in order to avoid the stationary red vehicle ahead. Figure 9 and Table 7 show that the participants exhibited an overall mean indicator time of 13.66 s (SD = 6.59 s). Female drivers exhibited slighter faster decisions to change lanes (M = 13.52 s, SD = 6.46 s) than the male drivers (M = 13.76 s, SD = 6.71 s). In order to test whether or not the difference in indicator time between female and male drivers is statistically significant, an Independent samples t-test was conducted and it shows that the difference is not statistically significant, t(302) = − 0.321, p = 0.748.

**Table 7:** Descriptive analysis of indicator time.

Indicator time (s)					
	Mean	SD	Min	Max	95% CI
Female	13.52	6.46	3.00	29.90	12.41–14.63
Male	13.76	6.71	3.15	36.10	12.75–14.77
Total	13.66	6.59	3.00	36.10	12.91–14.40

### Time to collision (TTC)

**Figure 10:**
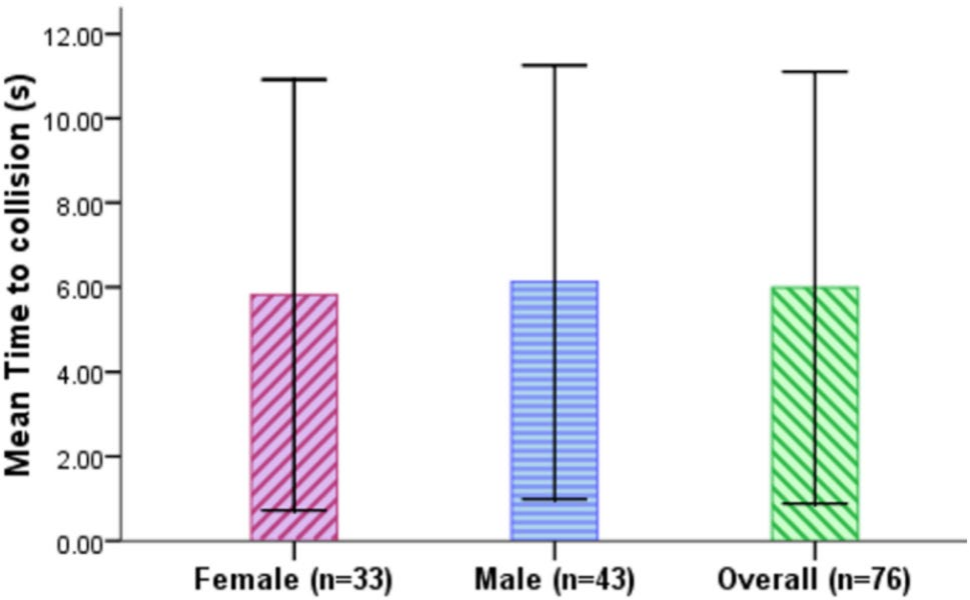
TTC time for female and male drivers (Error bars =  ± SD).

TTC is the time needed for the L3 AV to crash into the stationary red car ahead if it continued to drive at its current speed to the point at which it has completely changes to the adjacent lane and has successfully avoided the red car ahead. It measures the criticality of the drivers’ takeover where the smaller the value, the more critical the takeover. As Fig. 10 and Table 8 show, the participants exhibited an overall mean TTC of 5.99 s (SD = 5.11 s). Male drivers (M = 6.12 s, SD = 5.13) exhibited slightly longer TTC than the female drivers (M = 5.82, SD = 5.10 s). In order to test if the difference in the TTC among the two gender groups is significant, an Independent samples t-test is implemented. It revealed that the difference is not statistically significant, t(302) = − 0.514, p = 0.607.

**Table 8:** Descriptive analysis of TTC.

TTC (s)					
	Mean	SD	Min	Max	95% CI
Female	5.82	5.10	0.00	21.82	4.94–6.69
Male	6.12	5.13	0.00	25.81	5.35–6.89
Total	5.99	5.11	0.00	25.81	5.41–6.57

### Resulting acceleration

**Figure 11:**
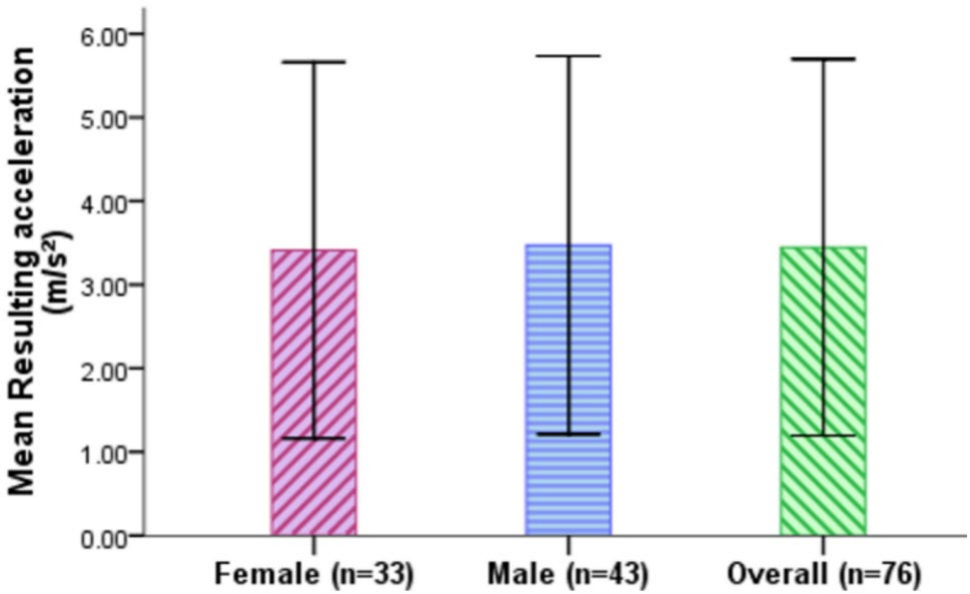
Resulting acceleration for female and male drivers (Error bars =  ± SD).

Resulting acceleration is calculated from the maximum longitudinal and lateral acceleration that the drivers generated during the takeover of control in L3 AVs (see Table 2). It measures the maximum force that the L3 AV transfers to the road during the takeover. The larger the value, the higher chance that the takeover is unstable. Figure 11 and Table 9 show that the participants exhibited an overall mean resulting acceleration of 3.45 m/s^2^ (SD = 2.25 m/s^2^). Female drivers (M = 3.41 m/s^2^, SD = 2.25 m/s^2^) generated slightly smaller resulting acceleration compared to the male drivers (M = 3.47 m/s^2^, SD = 2.26 m/s^2^). In regard to the effect of gender on the resulting acceleration, an Independent sample t-test showed that there was no significant effect of gender on the resulting acceleration, t(302) = − 0.234, p = 0.815.

**Table 9:** Descriptive analysis of resulting acceleration.

Resulting acceleration (m/s^2^)					
Female	Female	Female	Female	Female	Female
Male	Male	Male	Male	Male	Male
Total	Total	Total	Total	Total	Total

### Steering wheel angle

**Figure 12:**
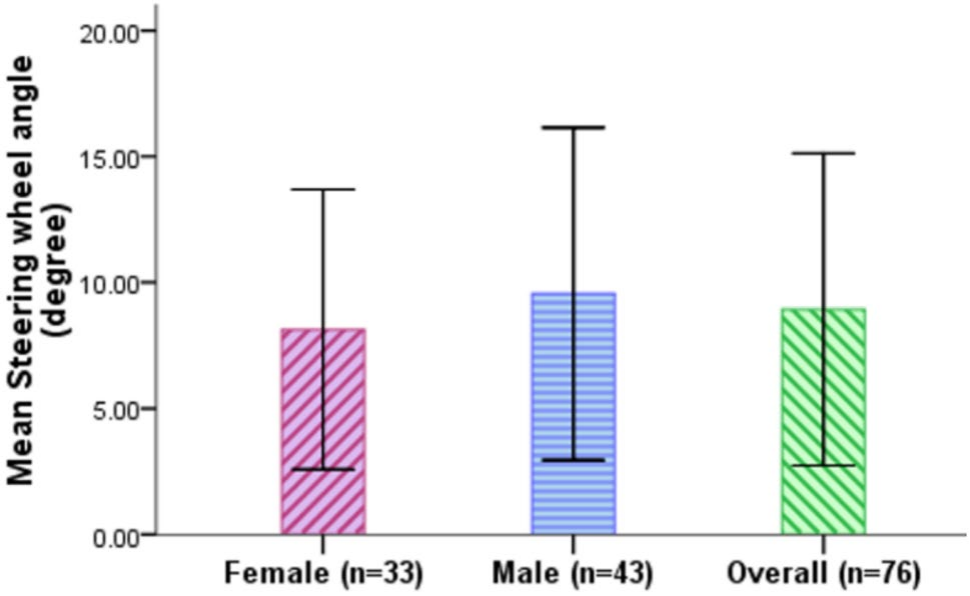
Steering wheel angles for female and male drivers (Error bars =  ± SD).

Steering wheel angle is the standard deviation in degrees of the angle to the centre line of the steering wheel. It measures the stability of the takeover, where a higher value reflects a less stable takeover. Figure 12 and Table 10 show that the participants had an overall mean steering wheel angle of 8.93° (SD = 6.19°). Female drivers exhibited a smaller steering wheel angle of 8.13° (SD = 5.55°) than the male drivers (M = 9.55°, SD = 6.60°).

**Table 10:** Descriptive analysis of steering wheel angle.

Steering wheel angle (°)					
	Mean	SD	Min	Max	95% CI
Female	8.13	5.55	1.77	31.47	1.95–4.48
Male	9.55	6.60	1.43	36.04	8.55–10.54
Total	8.93	6.19	1.43	36.04	8.23–9.63

An Independent samples t-test was performed to determine if there is a statistically significant difference in steering wheel angle between the female and male drivers, and the results revealed a significant effect of gender, t(302) = − 2.024, p = 0.044, Cohen's d = 0.231 with female drivers (M = 8.13°, SD = 5.55°) exhibiting significantly smaller steering wheel angle compared to the male drivers (M = 9.55°, SD = 6.60°), leading to a statistically significant difference of 1.41° (95% CI 0.04° to 2.78°).

## Discussion

This study has investigated drivers’ performance when taking over control from L3 AVs in different weather conditions. The effect of weather on drivers’ takeover performance has been reported in a separate publication^17^. The present study focuses on the effect of gender on drivers’ performance when taking over control from L3 AVs.

In terms of the strategies that female and male drivers adopted to react to the takeover request and to avoid the stationary red car ahead, the majority of both female and male drivers responded to the red car by only steering to the next lane without braking. This may be because the participants were provided with a relatively long lead time (20 s) to take over control of the vehicle and to avoid the potential collision with the red car ahead compared to those used in previous similar studies^7–9,14–16,19,20^. Moreover, Gold et al.^7^ found that the longer the lead time participants are provided with to reassume control of the L3 AV, the less likely it is that they would use the brakes during the takeover process. In addition, in clear weather, rain and snow conditions, the proportion of the drivers who responded to the stationary red car by both braking and steering to the next lane was higher among female drivers than male drivers. Also, female drivers exhibited a smaller percentage of hasty takeovers 12.8%, n = 17) compared to the male drivers (13.3%, n = 23). These findings indicate a slightly better takeover performance among female participants compared to male participants. Although the differences are not statistically significant, the findings may still support the notion that females are generally more cautious drivers and are sometimes more careful when responding to critical situations than males^29,30,42^. These findings are in accordance with those of a previous study by Crizzle et al.^43^. Although their study was not closely comparable to the current research, similar results were found that male drivers made slightly more driving mistakes compared to female, but the difference was not statistically significant.

In regard to the effect of gender on takeover performance in L3 AVs, this investigation was found that the female and male drivers exhibited similar performance in terms of most of the measurements adopted to quantify takeover performance. However, gender had a significant effect on reaction time, with female drivers exhibiting significantly faster reaction times (a significant difference of 0.17 s, 95% CI 0.002 s to 0.35 s) compared to the male drivers. Although the However, this finding differs from those of previous studies concerning gender differences in reaction time. For example, research by Blough and Slavin^44^ found that females had better performance than males in performing visual tasks, but their reaction time was slower than males. Jain et al.^45^ found that female participants exhibited slower reaction times to both visual and sound stimuli compared to male participants. A possible explanation is that, in the present study the reaction time is defined as the time between the moment that the L3 AV system issues the takeover request and the moment that drivers have completely switched to the manual driving position, which is the position where the drivers have put their hands on the steering wheel, feet on the pedals and eyes on the road. Before the moment that the L3 AV detects the stationary red vehicle and initiates a takeover request to the drivers, it was performing automated driving and the drivers were performing the non-driving related task (reading) and were completely disengaged from driving. Therefore, at the moment that they were suddenly asked by the L3 AV to take over control of the vehicle, they had little information about the current driving situation. Croson and Gneezy^42^ suggested that, when dealing with uncertain situations, females are more cautious and less confident compared to males, which may have resulted in a slightly faster movement to switch back to the manual driving position among female participants in this study, thus leading to a faster reaction time. Moreover, another possible reason could be that, as females are found to be more concerned about automated vehicles than males^33,36^, their higher level of concern may have led to a more eager desire to regain manual control of the vehicle, thus they exhibited faster reactions as soon as they perceived the takeover request issued by the L3 AV. Apart from their faster reaction times, female drivers were found to have significantly smaller steering wheel angles compared to male drivers, with a significant difference of 1.41° (95% CI 0.04° to 2.78°), which reflects a slightly more stable operation of the steering wheel during the takeover process and thus indicating better takeover performance. This is in accordance with the findings of previous studies about gender difference in terms of driving behaviour. Compared to males, female drivers exhibited more cautious and less risk-taking driving behaviour, and were more patient in urgent situations^29,30,42^.

Although females are found to have better takeover performance than males in the present study, the findings of previous studies focusing on the attitudes and opinions of automated vehicles have revealed that women are less positive and more concerned about automated vehicles and are less willing to own and use them compared to males^32–35^. A possible reason for the relatively negative attitudes towards automated vehicles among women could be due to the lack of confidence in believing that they are capable of using automated vehicles well. As Moè et al.^28^ argued, women drivers are sometimes less confident than men and may not be aware that they can drive well. However, the findings of the present study provide evidence suggesting that relatively less positive attitudes and perceptions among women towards automated vehicles did not mean they have worse performance when interacting with L3 AVs. On the contrary, they exhibited better takeover performance than their male counterparts in this study.

## Implications and recommendations

The findings of the present study provide several important implications for policy-makers, original equipment manufacturers (OEMs) and academics in terms of designing and facilitating user-friendly human–machine interactions in L3 AV. The results of this study showed that both gender groups showed similar takeover performance in most of the measurements of takeover, and female participants exhibited slightly better performance in terms of the reaction time, steering wheel angle and number of hasty takeovers. However, when operating the conventional vehicles, Moè et al.^28^ found that women are sometimes not able to recognise that they are good drivers and recommended that successful driving experience could potentially be helpful in enhancing their confidence in driving. This recommendation could also be applied to increase the confidence and acceptance of potential female end-users of L3 AVs. Also, an appropriate strategy for introducing L3 AV to end-users could be adopted. In such introduction strategies, the findings of relevant research and successful use cases of different demographic groups interacting with L3 AV could be used to help end-users to build a comprehensive and objective understanding of their capabilities in being able to operate and interact with the L3 AV smoothly and effectively. In addition, more opportunities for hands-on experience with the L3 AV could be offered to the potential end-users, which would not only enable them to build a realistic understanding of automated vehicles but also potentially help them to develop their confidence in the safe and comfortable usage of L3 AV. More teaching and demonstration sections in how to use L3 AV could be provided. As Abraham et al.^37^ suggested, unlike males who are always good at self-learning in using new in-vehicle technologies, female drivers could benefit more from being taught by others.

Moreover, in the present research, in order to achieve a completely disengagement from driving status for the participants in the L3 AV, participants from both groups were asked to perform the same mandatory reading tasks before being asked to take over control^19,46^. However, in reality, different end-users may tend to perform different activities while driving. For example, a common traffic violation for female drivers is applying cosmetics while driving^47,48^. For men, the more common problem is using mobile phones while driving^49^. Although such activities are illegal under current law when driving conventional vehicles, the forthcoming L3 AV could allow drivers to perform them when the car is driving in automated mode^3–6,18^. The different non-driving related tasks preferred by different end-users could potentially affect drivers’ takeover performance in L3 AVs. Such differences should be taken into account when designing and testing L3 AVs. For instance, the design of the car interiors of L3 AVs could provide a variety of facilities, such as, a foldable dining table, a compact dressing table, mobile phone holders and docking stations, in order to satisfy end users with different needs. And more research should be conducted to understand the impact of performing different non-driving related activities on the takeover performance of end-users from different demographic groups in L3 AVs. Finally, the results indicate that both female and male drivers exhibited higher numbers of hasty takeovers and CCEs in snowy and foggy conditions compared to clear weather and rainy conditions. As proposed elsewhere^17^, in such adverse weather conditions it would be safer to use cars equipped with Level 4 automation^6^ that can activate the safe mode by themselves even if the driver cannot react safely and effectively to a takeover request.

In summary, the following recommendations should be considered in order to facilitate a user-friendly human–machine interaction in L3 AVs for end-users with different needs.

- When introducing the L3 AV to end users, it is important to enable end-users to recognize that they can use and interact with L3 AV well.
- Providing hands-on experience and teaching sessions in using L3 AVs could enable end- users to build their confidence and deepen their understanding of L3 AVs.
- When designing L3 AVs, car interiors could be designed to support various types of non-driving related tasks during automated driving mode; for example, and providing foldable dining tables, compact dressing tables, mobile phone holders/docking stations.
- Further research is needed to investigate the influence of performing different non-driving related tasks on the takeover performance of end-users from different demographic groups.

## Conclusions

The present research aims to investigate the effect of gender on the takeover performance in L3 AVs. We found marked gender differences in terms of the performance of taking over control in L3 AVs. In general, women exhibited better takeover performance than men. Compared to the male participants, a smaller percentage hasty takeovers were recorded among female participants, although the difference is not statistically significant. In addition, female drivers exhibited significantly faster reactions to the takeover request initiated by the L3 AV system and significantly more stable operation of the steering wheel during the process of taking over control compared to male participants. The present research has created knowledge that is helpful in understanding the gender differences in interacting with L3 AVs from a performance perspective. The findings of this study also emphasise the importance of considering gender when conducting research into and designing automated vehicles, which in accordance with the argument that gender lens is required when assessing transportation systems and user behaviour^50^.

Although previous research has found that women in general have less positive attitudes towards automated vehicles compared to men, the findings of this study evidenced that women are able to use and interact with L3 AVs better than men. The implication of the findings as well as the recommendations proposed by the present study could be helpful to policymakers, OEMs and transport academics to potentially facilitate a more user-friendly design of human–machine interaction in L3 AVs.

While this study has yielded useful findings, there are still limitations. To begin with, this study examined participants’ takeover performance in L3 AVs using a driving simulator, which is an effective method for studying drivers’ interaction with in-vehicle technologies^12,13,17,31,46^. However, in real life, there will be safety critical consequences if drivers fail to take over control in time or properly in a level 3 automated vehicle. Therefore, there is still a need for future research to validate the results of the present study in real road conditions using an authentic full-scale level 3 automated vehicles^51^. The sample size was slightly imbalanced sample for male and females. Future work has been planned to explore the impact of gender by adopting an equal sample size and using an authentic full size automated vehicle in a real world situation. Due to the lack of similar research, the obtained effect sizes of the significant results of this study were not able to be compared to effect sizes reported in previous studies with similar features in order to interpret if they are comparable with, or smaller/larger than previous research^52^. However, the obtained effect sizes of this study could be important references for future studies of gender and vehicle automation in order to better understand, compare and interpret the effect size of the results. This research focused on the gender effect on takeover performance, future research could examine the impact of other important factors on people’s interaction with L3 AV, such as, people’s experience of using Advanced Driver Assistance Systems and their driving experience. In addition, this study asked drivers to perform a reading task during automated driving mode in L3 AVs; however, there is a potentially diverse range of other activities that could be undertaken by different end-users when travelling with L3 AVs in real life, such as applying make-ups and using mobile phones. Future research could explore gender difference in the requirements associated with non-driving related activities^18,53^ and test the influence of performing these tasks on drivers’ performance. Also, future research could also survey participants’ exposure to video driving or racing games and investigate how it impacts their interaction with automated vehicles. The present study quantitatively investigated female and male drivers’ interaction with L3 AVs, future research could qualitatively explore the gender difference in the requirements and preferences toward the human–machine interfaces in the L3 AVs^18,54,55^. Moreover, the dependent variables adopted in the present study mainly focused on measuring participants’ performance of retaking control from L3 AVs, future research could adopt other measurements to quantify drivers’ stress level, workload and motion sickness when interacting with the L3 AVs^11,31,56^. All participants in this study have used indicator lights to show an intended lane change after reassuming control from the automated driving systems. Failing to use indicators is regarded as careless and inconsiderate driving. Future research could use ‘Not using indicator light’ as a dependent variable to assess driving performance after taking over control from the automated vehicle. This study did not include any surrounding traffic in the L3 AV scenario apart from the red vehicle obstructing the lane ahead. Future research has been planned to specifically investigate the impact of surrounding traffic density on subjects’ performance and behaviour when taking over control from automated vehicles. Finally, the present study provided drivers with a lead time of 20 s to take over control of the vehicle and avoid a potential collision. This would require the L3 AV system to be able to detect the stationary red vehicle blocking the road ahead at a long distance in advance (268.2 m when travelling at 30mph and 536.4 m when travelling at 60mph). In order to achieve this, it is imperative to design and develop user-friendly automated vehicles that are cooperative and connected^12,18,57,58^.
